# Quality of life following liver transplantation: a comparative study between Familial Amyloid Neuropathy and liver disease patients

**DOI:** 10.1186/1471-230X-9-54

**Published:** 2009-07-15

**Authors:** Diogo Telles-Correia, Helena Cortez-Pinto, António Barbosa, Inês Mega, Estela Monteiro

**Affiliations:** 1 Department of Psychiatry, Faculty of Medicine, University of Lisbon, Portugal; 2 Psychiatric approach to Liver Transplanted Patients' Unit of Curry Cabral Hospital's Liver Transplantation Center, Lisbon, Portugal; 3 Department of Gastroenterology, Faculty of Medicine, University of Lisbon, Portugal

## Abstract

**Background:**

It has been demonstrated in many studies that quality of life can be improved after liver transplantation in patients with liver disease. Nevertherless quality of life improvement in specific groups of transplantated patients such as those with Familial Amyloid Polineuropathy hasn't yet been explored. The present study aimed to compare the change in quality of life following liver transplantation between patients with Familial Amyloid Polineuropathy (FAP) and patients with liver disease.

**Results:**

Patient's mental quality of life showed an improvement in all liver disease patients, and a worsening in FAP patients, resulting in a significant difference between the two groups. Regarding physical quality of life, although a similar improvement was seen in both groups, FAP patients had significantly less improvement than the sub-group of decompensated liver disease (Child-Pugh B and C).

**Conclusion:**

It is concluded that liver transplantation has a less beneficial impact in FAP patient's physical quality of life, probably because they are not so much disabled by their disease at the moment of liver transplantation. The lesser improvement in mental quality of life of FAP patients may be due to their particular psychological profile and greater expectations towards transplantation.

## Background

It has been demonstrated in many studies that quality of life can be improved after liver transplantation in patients with liver disease and that this improvement is present 6 months after transplantation [1-4].

Familial amyloid polyneuropathy (FAP) is an autossomal dominant multisystemic fatal disorder characterized by a progressive peripheral and autonomic neuropathy with neural and systemic amyloid deposits [6,7]. The disease is caused by a mutant gene in chromosome pair 18. The amyloid protein in type 1 FAP of Portuguese, Swedish and Japanese origin is the variant of transthyretin 8TTR, in which methionine is a substitute for valine at position 30 (TTR Met). More than 90% of this TTR Met 30 is produced by the liver and the rest by choroid plexus. The most consensual way to treat FAP is liver transplantation in the initial stage of the disease, to prevent neurological deterioration. Patients with FAP are almost asymptomatic when they are transplanted in contrast to other liver transplant candidates that usually have a disabling chronic liver disease [6,7]. FAP patients might have particular personality and psychiatric characteristics compared with liver transplanted patients [8]; furthermore, several studies have shown an improvement in survival and in the progression of the disease in FAP patients after liver transplantation [8-11]. However, to our knowledge, there are no prospective studies in the literature investigating FAP patient's quality of life after liver transplantation.

The aim of the present prospective study was to compare the change in quality of life after liver transplantation patients with FAP and liver disease.

## Methods

### 1) Participants

A group of 66 consecutive transplant candidates, attending the out-patient clinics of a Liver Transplantation Centre in Lisbon, were studied between March 1, 2006 and December 1, 2007. Written informed consent was obtained from all participants, and the study protocol was approved by the institutional review committee. These patients were assessed before transplantation and 6 months after being transplanted.

### 2) Quality of Life evaluation

We used the SF-36 Portuguese validated version, a self rating questionnaire developed by the Medical Outcome Trust [12], to investigate certain primary aspects of quality of life. The SF-36 has been widely used under a range of different medical conditions and shown to have adequate reliability and validity. The first four subscales refer to physical aspects, and the last four scales, mental aspects: Physical Functioning, Physical Role Limitation, Bodily Pain, General Health, Vitality, Social Functioning, Emotional Role Limitation and Emotional Well Being. Physical aspects mostly refer to physical capability to perform normal daily activities. The mental dimension mostly refers to social aspects of life the degree to which illness interferes with emotional well-being, and social roles. Total values were computed for physical and mental components of health-related quality of life by averaging the eight weighted subscales using the coefficients generated by Hays et al [13] in the Medical Outcomes Study.

### 3) Medical Evaluation

Diagnosis of chronic liver disease was done by a hepatologist. Child-Pugh classification was used to evaluate liver disease severity.

Diagnosis of FAP was done by a neurologist. To evaluate the severity of FAP a Portuguese classification was used [14]: Level 0 – asymptomatic; Level 1 – sensitive and/or dysautonomic symptoms without neurological signs; Level 2 – sensitive and/or dysautonomic symptoms + neurological signs (sensitive); Level 3 – sensitive and/or dysautonomic symptoms + neurological signs (sensitive-motor) in lower limbs with independent walking; Level 4 – neurological signs in lower and upper limbs (sensitive or motor) walking without help; Level 5 – neurological signs (sensitive or motor) in lower and upper limbs, in wheel chair; Level 6 – confined to bed.

### 5) Statistical Methods

Statistical analysis was done using the SPSS 13.0 for Windows^® ^software package. Descriptive data were presented in absolute frequencies, percentages, mean values, standard deviations.

We constructed a new variable (DIFQL) to measure the difference between quality of life measured 6 months after transplantation (QL6M) and before transplantation (QLBT), (DIFQL = QL6M-QLBT). This new variable was used to measure the change in mental health (mentalDIFQL) and physical health (physicalDIFQL) components of quality of life, after liver transplantation.

Once the two groups are very different between them, comparing absolute values wouldn't reflect the real difference between the groups. Thererefore, a new variable that reflects the actual variation of quality of life after transplantation was created (DIFQL).

## Results

### 1) Demographic and Medical Data

Regarding medical diagnosis, 20 patients had FAP and 46 had chronic liver disease (CLD). Among the patients with chronic liver disease, 13 had Alcoholic Liver Disease (ALD), 7 hepatitis C associated cirrhosis (HCAC), 2 had hepatocellular carcinoma (HCC), 2 Primary Biliary Cirrhosis, 1 Familial Progressive Cholestasis, and the others had Mixed Diagnosis (7 ALD + HCC; 7 HCAC + HCC; 2 HCAC+HCC+ALD; 1 HCC+ Virus B Liver Disease; 1 Hemochromatosis + LC), unknown cause 3 (Tables 1, 2, 3 and 4).

**Table 1:** Medical Diagnosis

Medical Diagnosis	N
**FAP**	20
**Chronic Liver Diseases**	
Alcohol Liver Disease (ALD)	13
Hepatitis C associated Cirrhosis	7
Hepatocellular carcinoma (HC)	2
Primary Biliary Cirrhosis	2
Familial Progressive Cholestasis	1
Mixed Diagnosis	18
Unknown Cause	3

**Table 2:** Differences between FAP group and Liver disease Group concerning DIFQL

	FAP group n = 20	Liver Disease Group n = 46	Z	p
mentalDIFQL	-7.08	18.47	z = -3.04	p = .002
physicalDIFQL	8.00	16.76	z = -1,348	p = .178

**Table 3:** Differences between FAP group and Child Pugh A Liver disease Group concerning DIFQL

	FAP group n = 20	Moderate Liver Disease Group n = 22	Z	p
mentalDIFQL	-7.08	11.9524	-2.056	P = .040
physicalDIFQL	8.00	6.3810	-1,348	p = .178

**Table 4:** Differences between FAP group and Child Pugh B/C Liver disease Group concerning DIFQL

	FAP group n = 20	Severe Liver Disease Group n = 24	Z	P
mentalDIFQL	-7.08	26,6522	z = -4.21	p = .000
physicalDIFQL	8.00	28,4783	z = -3.32	p = .000

In FAP group (n = 20), 11 patients were male, and mean age was 31.8 years. Seven patients belonged to Level 1 FAP, with symptoms but without neurological signs, and the other thirteen to the Level 2 and 3, with neurological signs (sensitive/motor) but walking without help.

In the liver disease group (n = 46), 32 were male and mean age was 52.4 years. Twenty one patients belonged to Child-Pugh class A, 14 to B class and 9 to C class.

The main reasons by which non PAF Child-Pugh class A patients were transplantated were liver cancer and severe liver disease complications (such as hemorrhagic complications).

The mean hospital stay was 20.3 days. Within the whole sample, 26 patients had some kind of complication along the 6 first months after transplantation (15 had an infection, 4 a vascular complication, 5 a biliar complication, 3 an acute rejection). Patients that died after transplantation or had to be retransplantated were excluded from the study.

No statistical associations where found between demographic characteristics and presence of complications, and quality of life scores.

### 2) Quality of Life Differences

In the FAP group, the absolute scores for quality of life's mental and physical components were, before transplantation 63.00 and 58.00, and after transplantation 55.92 and 66.00. In the non FAP group, the absolute scores for quality of life's mental and physical components were, before transplantation, 60.00 and 48.00 and after transplantation 66.76 and 67.47.

Compaired to normative values of quality of life (for healthy pearsons) (physical component:73.08; mental component:67.14) [15] the scores of the FAP and nonFAP groups in the pre-transplantation period were significantly lower. Twelve months after transplantation there were no significant differences between non FAP group and normative data, but quality of life mental component was significantly lower for FAP patients, compaired to normative data (p = .002).

For FAP group, mental DIFQL was -7.08 and physical DIFQL was 8.00, for liver disease's group mental DIFQL was 18.47 and physical DIFQL 16.76.

We compared mental DIFQL and physical DIFQL between FAP group and liver disease group. We found that there was a statistical significant difference between the two groups for mental DIFQL (z = -3.04, p = .002) but not for physical DIFQL (z = -1.348, p = .178).

Considering that the absence of difference in physicalDIFQL, might be due to the paucity of symptoms in Child-Pugh class A patients, the liver disease group was divided into 2 groups: A and B+C, and compared each of these subgroups with the FAP. By doing so, a significant difference for mentalDIFQL (z = -2.056, p = .040) was found between FAP patients and Child-Pugh A, and a significant difference for mentalDIFQL (z = -4.21, p = .000) and for physicalDIFQL (z = -3.321, p = .000) between FAP patients and severe liver disease patients (Child-Pugh B/C).

## Discussion

Most studies found an improvement in mental and physical quality of life components after liver transplantation [1-4]. However the great majority of these studies evaluated decompensated liver disease patients, with a very disabling disease at the time of liver transplantation.

To our knowledge, this is the first study evaluating mental and physical quality of life FAP patients after liver transplantation, and in fact this is a very particular group of liver transplanted patients. FAP patients have a genetic disease, invariably fatal without treatment, and are now being transplanted at a very early stage, with a paucity of symptoms [6,7]. There are some studies about the clinical progression of these patients after transplantation, the majority demonstrating a slowing in the progression of neurological symptoms [8-11].

The fact that liver transplantation had a less beneficial impact in mental quality of FAP patients is difficult to explain. It cannot be explained by the paucity of symptoms that FAP patients have before transplantation, since when comparing them with liver disease patients with few symptoms, such as Child-Pugh A, they still have a worse evolution of their mental quality of life, although the improvement in physical quality of life was similar.

One possible explanation could relate to a particular psychological profile of these patients. Telles-Correia 2008 et al found that there was a high prevalence of psychiatric diagnosis (mostly depression and anxiety) in familial amyloid polyneuropathy patients waiting for transplantation and that these patients had a different personality characteristics comparing to other liver transplant candidates [7]. This might be due to the emotional stress of being exposed to a chronic sensation of being carrier of a fatal genetic disease [16]. This chronic emotional stress added to a maintained exposure to doubt and uncertainty feelings might explain the development of great expectations towards liver transplantation, and partially explain the difference towards mental quality of life changes found in the present study. These hypotheses must be confirmed by other studies.

On the other hand, our findings that liver transplantation has a less beneficial impact in physical component of quality of life in FAP patients is easier to explain, because FAP patients are not so much disabled by their disease at the moment of liver transplantation, than decompensated liver disease patients.

One of the main weaknesses of the present study is the small number of FAP patients included, what relates to the difficulty of doing a prospective study in such a rare disease. More studies in this area are needed to confirm our findings and further explore our knowledge of the FAP psychological profile, in order to better deal with these patients and improve their quality of life after liver transplantation.

## Conclusion

We concluded in this prospective study that liver transplantation has a less beneficial impact in FAP patient's physical quality of life, probably because they are not so much disabled by their disease at the moment of liver transplantation. The lesser improvement in mental quality of life of FAP patients may be due to their particular psychological profile and greater expectations towards transplantation.

## Competing interests

I declare that none of the authors has any financial or non-financial competing interests.

## Authors' contributions

DTC conceived of the study, and participated in its design and coordination and helped to draft the manuscript. HCP conceived of the study, and participated in its design and coordination and helped to draft the manuscript. AB conceived of the study, and participated in its design and coordination and helped to draft the manuscript. IM participated in the sequence alignment and drafted the manuscript. EM participated in the sequence alignment and drafted the manuscript. All authors read and approved the final manuscript.

## Pre-publication history

The pre-publication history for this paper can be accessed here:

http://www.biomedcentral.com/1471-230X/9/54/prepub
